# Temperature effects on an InGaP (GaInP) ^55^Fe X-ray photovoltaic cell

**DOI:** 10.1038/s41598-017-05181-8

**Published:** 2017-07-10

**Authors:** S. Butera, M. D. C. Whitaker, A. B. Krysa, A. M. Barnett

**Affiliations:** 1 Semiconductor Materials and Devices Laboratory, School of Engineering and Informatics, University of Sussex, Brighton, BN1 9QT UK; 2 EPSRC National Centre for III-V Technologies, University of Sheffield, Mappin Street, Sheffield, S1 3JD UK

## Abstract

This paper investigates the effects of temperature on an InGaP (GaInP) ^55^Fe X-ray photovoltaic cell prototype for a radioisotope microbattery (also called a nuclear microbattery). An In_0.5_Ga_0.5_P p-i-n (5 μm i-layer) mesa photodiode was illuminated by a standard 206 MBq ^55^Fe radioisotope X-ray source and characterised over the temperature range −20 °C to 100 °C. The electrical power output of the device reached its maximum value of 1.5 pW at a temperature of −20 °C. An open circuit voltage and a short circuit current of 0.82 V and 2.5 pA, respectively, were obtained at −20 °C. While the electrical power output and the open circuit voltage decreased with increasing temperature, an almost flat trend was found for the short circuit current. The cell conversion efficiency decreased from 2.1% at −20 °C to 0.7% at 100 °C.

## Introduction

Nuclear microbatteries^1, 2^ that convert the energy from photons/particles emitted by radioactive materials into electrical energy are attractive for many long life (>10 year) applications such as microelectromechanical systems (MEMS), implantable medical devices, and military equipment^3–5^. High energy density and small size are among the other advantages of such a type of power supply. Nuclear microbatteries can also be used under extreme conditions (including hot and cold temperatures, extreme pressures, mechanical shocks and vibration) for specialist applications in aeronautics and astronautics, deep ocean exploration, and instrumentation for hot and cold deserts. The effect of operating temperature on microbattery photovoltaic cell performance is of critical importance for many target applications; temperature can significantly affect the voltaic cell’s performance. To enable a wider operating temperature range, wide bandgap semiconductor materials are frequently used as the converter material. Wide bandgap materials also bring a further benefit of improved conversion efficiency compared to alternative narrower bandgap material such as silicon, since conversion efficiency increases linearly with bandgap^6^. Various wide bandgap materials have been considered for use in microbatteries, some with more success than others. GaN is a wide bandgap semiconductor (bandgap of 3.4 eV) that has attracted particular research attention for nuclear microbatteries. Betavoltaic microbatteries made of GaN detectors with a ^63^Ni radioactive source have been proven by Cheng *et al*.^7, 8^: the ^63^Ni-GaN microbattery with the best performances showed an open circuit voltage of 1.64 V, a short circuit current density of 14 nA/cm^2^ and a maximum output power density of 12.2 nW/cm^2^ ^8^. Another material that has been shown to be very useful in a nuclear microbattery is SiC. An highly efficient ^33^P-SiC betavoltaic cell has been demonstrated by Eiting *et al*.^9^ with an open circuit voltage of 2.04 V, a short circuit current density of 2000 nA/cm^2^ and maximum output power density of 2100 nW/cm^2^. A ^63^Ni-SiC betavoltaic microbattery has been reported by Chandrashekhar *et al*.^6^, such power supply presented an open circuit voltage of 0.72 V and a short circuit current density of 16.8 nA/cm^2^. Diamond nuclear microbatteries have been also successfully produced by Bormashov *et al*.^10^, and recently Delfaure *et al*.^11^ reported a single crystal diamond membrane for use as a betavoltaic cell. The cell was tested with a 20 keV electron beam showing an open circuit voltage and a short circuit current of 1.85 V and 7.12 μA, respectively. Nuclear microbatteries that use GaAs as converter layer have also been developed^12, 13^; Chen *et al*.^12^ reported a ^63^Ni-GaAs microbattery with an open circuit voltage of 0.3 V and a short circuit current density of 28 nW/cm^2^, whilst Wang *et al*.^13^ conducted a temperature dependence study of a GaAs cell under the illumination from ^63^Ni and ^147^Pm radioactive sources (d*V*_*OC*_/d*T* were −5.30 mV/°C and −4.90 mV/°C for ^63^Ni-GaAs and ^147^Pm-GaAs microbatteries, respectively). A tritium AlGaAs microbattery has been reported in ref. 14, such a microbattery presented an open circuit voltage of 0.47 V, a short circuit current density of 937 nA/cm^2^, and a maximum output power density of 259 nW/cm^2^. Al_0.52_In_0.48_P (bandgap of 2.31 eV)^15^ has also recently been reported for use in a betavoltaic cell, a temperature study of the key ^63^Ni-Al_0.52_In_0.48_P cell parameters showed that the best microbattery performances were reached at −20 °C (the lowest temperature analysed): open circuit voltage, short circuit current density and a maximum output power density of 0.52 V, 2 nA/cm^2^, and 0.2 nW/cm^2^ were observed, respectively. It should be noted that values reported for the different materials depend in part on the activity of the radioactive material being used to illuminate the cells.

Because of the high energy per Curie of radioisotope alpha particle and beta particle sources, much work has concentrated on analysis of alpha- and beta- voltaic cell structures (with more focus on betavoltaic microbatteries, since alphavoltaic microbatteries suffer radiation damage from the alpha particles)^16^. An alternative to alpha- and beta- voltaic devices is the X-ray photovoltaic device. The use of a radioisotope X-ray source compared with a radioisotope beta particle source further reduces the device damage risk; soft X-ray sources (Photon energies < 10 keV) can be also easily shielded providing safe working conditions, and are readily commercially available off the shelf. For these reasons, the electrical performance of X-ray photovoltaic microbatteries have been recently studied by Butera *et al*. using GaAs, AlGaAs, and AlInP detectors^17–19^ over a broad range of temperatures.

Another semiconductor that can be potentially very useful for microbattery technology is InGaP. In_0.5_Ga_0.5_P has a direct bandgap of ~1.9 eV at room temperature^20–22^ and can be grown, with high crystalline quality (low defect concentration) nearly lattice matched with GaAs. Due to its wide bandgap, In_0.5_Ga_0.5_P would experience lower thermally generated leakage currents than narrower bandgap devices of the same geometry. Furthermore, it would be able to work at high temperatures with minimal loss of performance. These characteristics, coupled to the expected high conversion efficiency and the relatively low production costs (since it can be grown on commercial GaAs substrates using common growth methods), make In_0.5_Ga_0.5_P an attractive choice for nuclear microbatteries. Previously, InGaP has been successfully used in an alpha-voltaic microbattery (coupled to ^241^Am or ^210^Po alpha-particle radioactive sources) by Cress *et al*.^23^ and a beta-voltaic microbattery (tritium based direct conversion microbattery) by Cabauy *et al*.^24, 25^.

The exceptional performances of InGaP/GaAs based structures can be also used in a variety of electronics and optoelectronics applications, including high electron mobility transistors (HEMTs), heterojunction bipolar transistors (HBTs), red emitting VCSEL and tandem solar cell^26–29^, at different temperatures. Huang *et al*.^26^ reported results of temperature dependent DC characteristics, minimum noise figure and associated gain of low noise In_0.49_Ga_0.51_P/In_0.15_Ga_0.85_As/GaAs pseudomorphic heterojunction bipolar transistors (PHEMTs) over the temperature range 300 K to 450 K; whilst Lin *et al*.^27^ studied the stabilities of the dc current gain, ideality factor, and offset voltage against variations in temperature (25–45 °C) and currents for Ga_0.5_In_0.5_P/GaAs heterojunction bipolar transistors (HBTs). VCSELs in the visible red spectrum (emission wavelength ranges from approximately 650 nm to 690 nm) were proven by Wipiejewski *et al*.^28^ using InGaP based devices over the temperature range 20 °C to 85 °C: the structure, grown on GaAs substrates, has an active layer constituted by three InGaP quantum wells and Bragg mirrors constituted by AlGaAs/AlAs multilayer structures. Feteha *et al*.^29^ reported Ga_0.49_In_0.51_P/GaAs cascade solar cells with a p^+^/n^+^ GaAs tunnel junction; the variation of the photovoltage, photocurrent, fill factor, efficiency, current–voltage characteristics and spectral response under different temperatures (25–75 °C), and light intensity values (1–40 sun), were investigated. In all these applications, the knowledge of material properties such as diffusion length, lifetime and minority electron mobility is essential; the temperature dependence of these properties has been reported by Schultes *et al*.^30^, over the temperature range 5 K to 300 K.

In this paper, an In_0.5_Ga_0.5_P X-ray photovoltaic cell is reported for the first time. The effect of temperature on the key cell parameters such as open circuit voltage, short circuit current, maximum output power, and conversion efficiency were investigated over the temperature range −20 °C to 100 °C.

## Materials and Methods

### Device structure and X-ray source

The In_0.5_Ga_0.5_P structure was grown on a (100) n-GaAs substrate by metalorganic vapour phase epitaxy using trimethylgallium, trimethylindium, arsine and phosphine as precursors and hydrogen as a carrier gas. For n-type and p-type doping, disilane and dimethylzinc were used, respectively. The epitaxial surface of the substrate had a miscut angle of 10° towards <111> A to avoid the spontaneous long-range ordering in the group III sublattice and related effects on the bandgap^31, 32^. First, an n-GaAs buffer layer was grown, and this was subsequently followed by n-In_0.5_Ga_0.5_P, nominally undoped i-In_0.5_Ga_0.5_P and p-In_0.5_Ga_0.5_P layers with thicknesses of 0.1 µm, 5 µm and 0.2 µm, respectively. The doped layers had a carrier concentration of 2 × 10^18^ cm^−3^. The structure was completed with a 0.01 µm thick highly doped (1 × 10^19^ cm^−3^) p-GaAs contact layer to ensure a good Ohmic contact. Chemical wet etching techniques were used to fabricate a 400 μm diameter In_0.5_Ga_0.5_P mesa photodiode; a 1:1:1 K_2_Cr_2_O_7_:HBr:CH_3_COOH solution was used followed by a 10 s finishing etch in 1:8:80 H_2_SO_4_:H_2_O_2_:H_2_O solution. Sidewall passivation techniques on the processed mesa In_0.5_Ga_0.5_P device were not used. Ti/Au (20 nm/200 nm) and InGe/Au (20 nm/200 nm) contacts were deposited on top of the GaAs top layer and onto the rear of the GaAs substrate to form the Ohmic top and rear contacts, respectively. The top Ohmic contact had an annular shape that covered 33% of the photodiode surface. The layers’ sequence, material compositions, thicknesses and doping types and levels in the fully fabricated structure are summarised in Table 1.

**Table 1:** Layer details of the In_0.5_Ga_0.5_P photodiode.

Layer	Material	Thickness (μm)	Dopant	Dopant Type	Doping density (cm^−3^)
1	Ti	0.02			
2	Au	0.2			
3	GaAs	0.01	Zn	p^+^	1 × 10^19^
4	In_0.5_Ga_0.5_P	0.2	Zn	p^+^	2 × 10^18^
5	In_0.5_Ga_0.5_P	5	undoped		
6	In_0.5_Ga_0.5_P	0.1	Si	n^+^	2 × 10^18^
7	Substrate n^+^ GaAs				
8	Au	0.2			
9	InGe	0.02			

**Figure 1:**
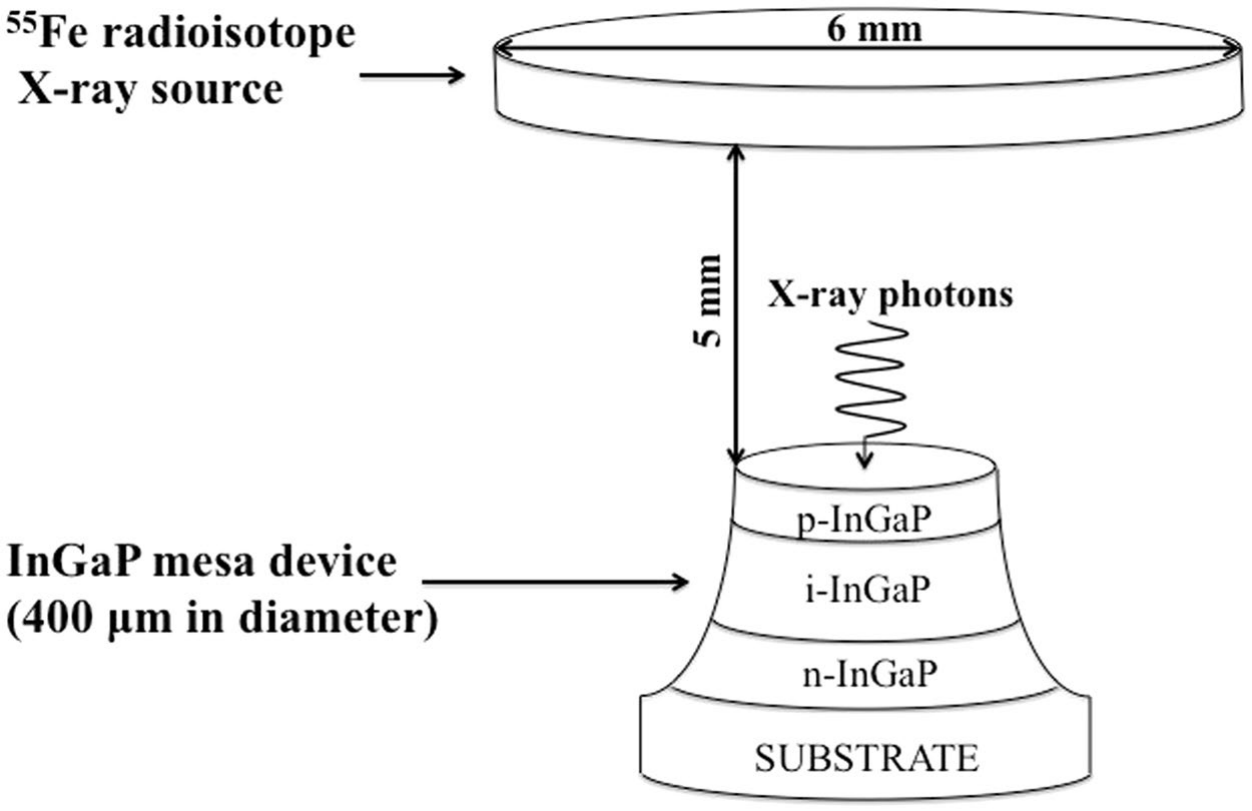
Schematic geometry of the ^55^Fe X-ray photovoltaic In_0.5_Ga_0.5_P cell; the figure is not drawn to scale.

A 206 MBq ^55^Fe radioisotope X-ray source (Mn Kα = 5.9 keV, Mn Kβ = 6.49 keV) was used to illuminate the 400 μm diameter In_0.5_Ga_0.5_P mesa photodiode. The X-ray emitter was placed 5 mm away from the top surface of the detector. Figure 1 shows the schematic geometry of the source and detector.

The In_0.5_Ga_0.5_P device’s X-ray quantum efficiency was computed using the Beer-Lambert law and assuming complete charge collection in the p and i layers. X-ray quantum efficiencies (*QE*_*NC*_) of 53% at 5.9 keV and 44% at 6.49 keV were obtained for the areas of the photodiode not covered by the top contact; whilst quantum efficiencies (*QE*_*C*_) of 44% at 5.9 keV and 38% at 6.49 keV were found for the areas covered by the top contact. In_0.5_Ga_0.5_P total quantum efficiencies (*QE*) were calculated from a weighted sum of *QE*_*NC*_ and *QE*_*C*_: total quantum efficiencies of 50% (*QE*_*Kα*_) and 42% (*QE*_*Kβ*_) were obtained at X-ray energies of 5.9 keV and 6.49 keV, respectively. The linear attenuation coefficients used in the *QE* calculations were 0.145 μm^−1^ and 0.112 μm^−1^ at 5.9 keV and 6.49 keV, respectively^33, 34^; these values are higher than for many other semiconductors such as Si, GaAs, and Al_0.52_In_0.48_P^33, 35^.

### Theoretical calculations

The incident power (*P*_*i*_) on the ^55^Fe X-ray photovoltaic In_0.5_Ga_0.5_P cell was calculated using equation 1:

1 $${P}_{i}=\frac{A}{2}\frac{{A}_{InGaP}}{{A}_{Fe}}(E{m}_{K\alpha }{T}_{K\alpha }5900+E{m}_{K\beta }{T}_{K\beta }6490)$$

where *A* was the activity of the ^55^Fe radioactive source (206 MBq), *A*_*InGaP*_ was the area of the In_0.5_Ga_0.5_P detector (0.13 mm^2^), *A*_*Fe*_ was the area of the ^55^Fe radioactive source (28.27 mm^2^), *Em*_*Kα*_ and *Em*_*Kβ*_ were the emission probabilities of Mn Kα and Mn Kβ X-rays from the ^55^Fe radioisotope X-ray source (0.245 and 0.0338, respectively^36^), *T*_*Kα*_ and *T*_*Kβ*_ were the transmission probabilities of Mn Kα and Mn Kβ X-rays through the 0.25 mm radioisotope X-ray source’s Be window (0.576 and 0.667, respectively^33, 35^). In equation 1, the activity of the ^55^Fe radioactive source was halved because half of the X-ray photons were lost since they were emitted upwards i.e. away from the X-ray photovoltaic device. *P*_*i*_ was found to be 72 pW.

The number of photons per second emitted in any direction by the ^55^Fe radioisotope X-ray source was 5.7 × 10^7^, this number was calculated knowing the activity of the source (206 MBq) and the emission probabilities of Mn Kα and Mn Kβ X-rays from ^55^Fe (0.245 and 0.0338, respectively^36^). Of these 5.7 × 10^7^ photons per second, only half were emitted in the direction of the device. Of these 2.9 × 10^7^ photons per second, only 1.7 × 10^7^ were transmitted through the X-ray source’s Be window (0.25 mm thick). The ratio between the area of the In_0.5_Ga_0.5_P device (0.13 mm^2^) and the area of the radioactive ^55^Fe source (28.27 mm^2^) was calculated to be 0.0044. The number of photons incident on the detector (7 × 10^4^ s^−1^) was estimated by multiplying 0.0044 for the number of photons per seconds transmitted through the X-ray source’s Be window (1.7 × 10^7^ s^−1^). The number of photons per seconds absorbed in the devices (4 × 10^4^ s^−1^) was calculated by multiplying the number of photons per second on the devices for the total quantum efficiency values calculated in section “Device structure and X-ray source”.

## Results and Discussion

The ^55^Fe X-ray photovoltaic In_0.5_Ga_0.5_P cell was placed inside a TAS Micro MT climatic cabinet with a dry nitrogen atmosphere (relative humidity < 5%). Dark and illuminated current characteristics as a function of forward applied bias (from 0 V to 1 V in 0.01 V increments) were measured using a Keithley 6487 picoammeter/voltage source over the temperature range −20 °C to 100 °C. The uncertainty associated with each current reading was 0.3% of its value plus 400 fA, while the uncertainty associated with the applied biases was 0.1% of their values plus 1 mV^37^. Dark and illuminated current characteristics as function of reverse applied bias were also obtained. Because of the low dark currents experienced by the In_0.5_Ga_0.5_P device, the measurement of any dark current displacements was important such to evaluate more accurately the X-ray induced current; therefore, upwards and downwards scans were performed and no hysteresis in the measurements was observed (the voltage scan rate was 13 sV^−1^, the voltage range was 0 V to 15 V).

**Figure 2:**
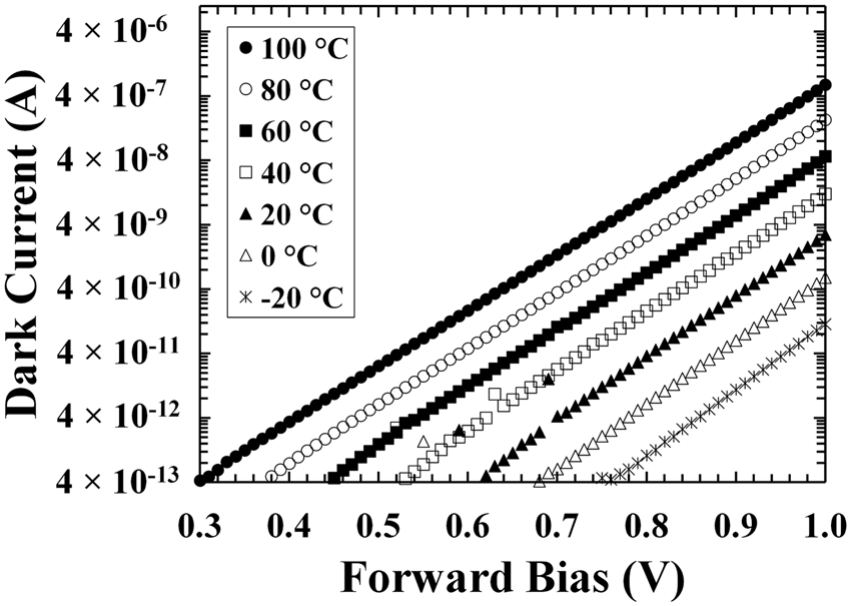
Dark currents as a function of applied forward bias for the In_0.5_Ga_0.5_P device. The temperatures studied were 100 °C (filled circles), 80 °C (empty circles), 60 °C (filled squares), 40 °C (empty squares), 20 °C (filled triangles), 0 °C (empty triangles), and −20 °C (stars).

Dark current characteristics as a function of forward bias for the In_0.5_Ga_0.5_P device at different temperatures are shown in Fig. 2.

**Figure 3:**
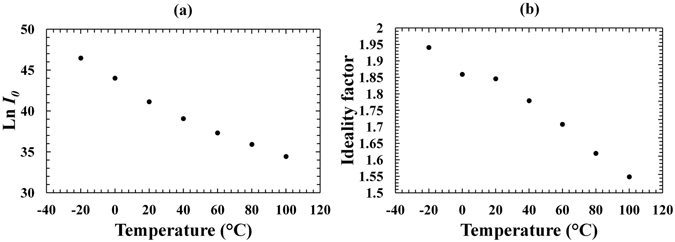
(**a**) The natural logarithm of the saturation current as a function of temperature for the In_0.5_Ga_0.5_P device. (**b**) The ideality factor as a function of temperature for the In_0.5_Ga_0.5_P device.

From −20 °C to 100 °C, an increase in the dark current through the device can be seen in Fig. 2; this could be due to the greater thermal energy available at higher temperatures, changes in series resistance could also contribute to the observed increase. The observed dependence between dark current and the applied forward bias followed the expected relationship for a simple p-n diode; such relationship is summarised in equation 2.

2 $$I={I}_{0}\exp \{qV/nkT\}$$

where *I*_*0*_ is the saturation current, q is the electric charge, *n* is the ideality factor, k is the Boltzmann constant, and *T* is the temperature^38^. The values of *I*_*0*_ and *n* for the In_0.5_Ga_0.5_P photodiode were experimentally estimated at each temperature: equation 2 was linearised as ln *I* = A + B*V*, with A = ln *I*_*0*_ and B = q(*n*k*T*)^−1^, and a linear least squares fitting was used. Figure 3a,b show the logarithm of the measured saturation current and the calculated ideality factor as functions of temperature, respectively.

In Fig. 3a, the natural logarithm of the saturation current decreased at increased temperatures. The observed decrease was 12.03 ± 0.04 (corresponding to a decrease in saturation current, *I*_*0*_, of 0.001 pA) between −20 °C and 100 °C. Making the simple assumption that the temperature dependence of the natural logarithm of the saturation current was proportional to (−*E*_*g*_ */2kT*)^17, 19^, the expected decrease was 14.66 (corresponding to a decrease in saturation current, *I*_*0*_, of 0.04 pA). The disagreement between the measured and the expected values may have been due to the temperature dependence of properties such as effective density of states, depletion width, and minority electron lifetime^14^.

Figure 3b shows the ideality factor which increased with decreasing temperature. At each temperature, an ideality factor >1.5 was estimated, indicating that the generation-recombination mechanism was dominant over the diffusion mechanism. The higher value of ideality factor observed at lower temperatures (1.941 ± 0.004 at −20 °C c.f. 1.5488 ± 0.0011 at 100 °C) may be attributed to the lower contribution of the diffusion current at lower temperatures^38^. Other semiconductor materials such as GaAs and AlInP present a similar dependence between ideality factor and temperature^10, 39^.

**Figure 4:**
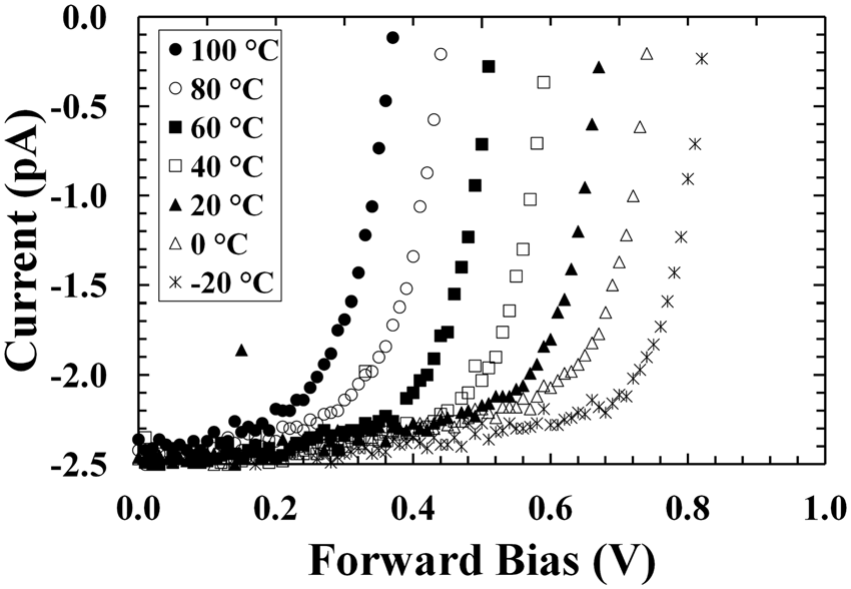
Current as a function of applied forward bias for ^55^Fe X-ray photovoltaic In_0.5_Ga_0.5_P cell. The temperatures studied were 100 °C (filled circles), 80 °C (empty circles), 60 °C (filled squares), 40 °C (empty squares), 20 °C (filled triangles), 0 °C (empty triangles), and −20 °C (stars).

Illuminated current characteristics as a function of forward bias for the ^55^Fe X-ray photovoltaic In_0.5_Ga_0.5_P cell at different temperatures are shown in Fig. 4.

**Figure 5:**
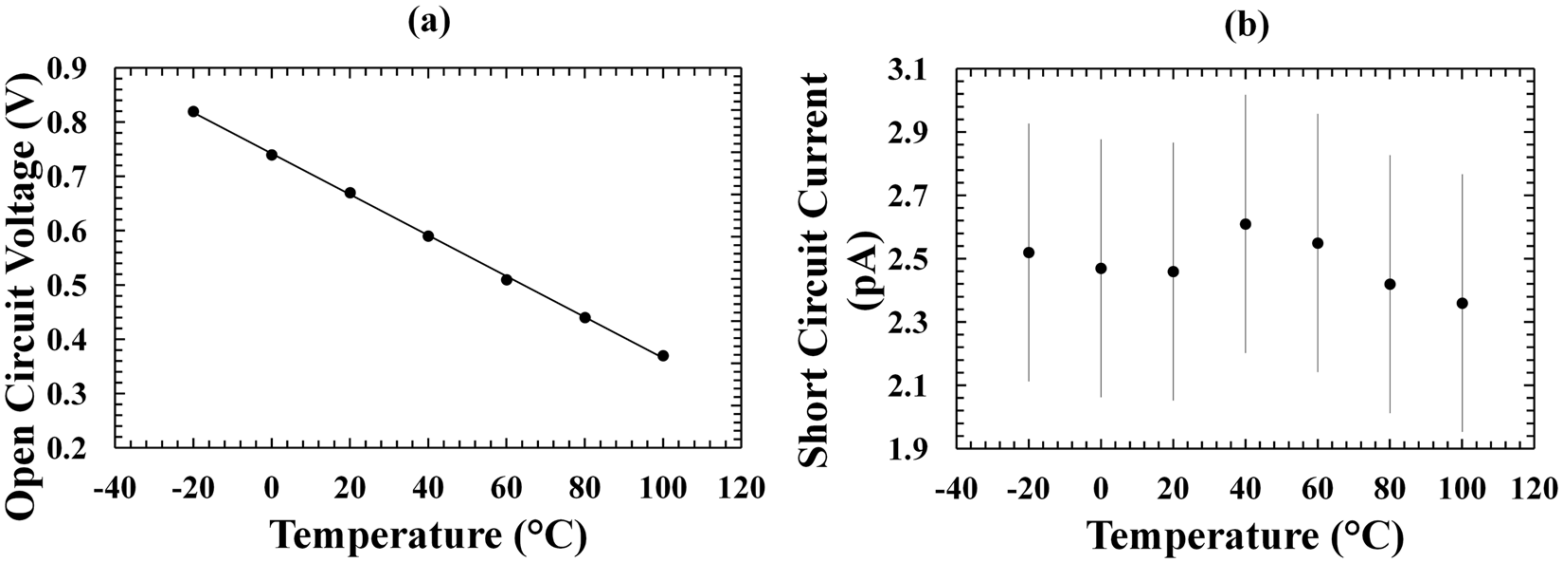
(**a**) Open circuit voltage as a function of temperature for the ^55^Fe X-ray photovoltaic In_0.5_Ga_0.5_P cell. Also shown is the line of the best fit computed by linear least squares fitting on the experimental data. (**b**) Short circuit current as a function of temperature for the ^55^Fe X-ray photovoltaic In_0.5_Ga_0.5_P cell.

As can be seen in Fig. 4, the softness in the knee of the measured current as a function of applied forward bias decreased with increased temperature. The interception points of the experimental curves on the horizontal and vertical axes gave the experimental values of the open circuit voltage (*V*_*OC*_) and the short circuit current (*I*_*SC*_), respectively. Figure 5a,b show the experimental values of the open circuit voltage and the short circuit current as functions of temperature, respectively.

A linear relationship between open circuit voltage (*V*_*OC*_) and temperature was observed, with the open circuit voltage reaching its maximum value of 0.82 V at −20 °C, as shown in Fig. 5a. The same figure also shows the linear least squares fit that was performed on the experimental data: *V*_*OC*_ = −A*T* + B where A = (0.00370 ± 0.00004) V°C^−1^, B = (0.742 ± 0.002) V. The open circuit voltage values achieved with the ^55^Fe X-ray photovoltaic In_0.5_Ga_0.5_P cell are higher than those reported with an GaAs ^55^Fe radioisotope microbattery^17^ but lower than those reported with an Al_0.52_In_0.48_P ^55^Fe radioisotope microbattery^19^, at the same temperatures. At −20 °C, for example, an open circuit voltage of 0.82 V is reported here, whilst values of 0.3 V and 0.97 V were demonstrated for the 10 μm i-layer GaAs and 2 μm i-layer Al_0.52_In_0.48_P ^55^Fe radioisotope microbatteries, respectively. An open circuit voltage of 1.7 V would be expected from a 5 μm i-layer Al_0.52_In_0.48_P device. These results can be explained considering that In_0.5_Ga_0.5_P has a bandgap higher than GaAs but lower than Al_0.52_In_0.48_P (at room temperature, the bandgap of Al_0.52_In_0.48_P, In_0.5_Ga_0.5_P and GaAs are ∼2.3 eV^40^, ∼1.9 eV^20–22^ and ∼1.42 eV^41^, respectively). The open circuit voltage is proportional to the logarithm of the ratio between generated current and saturation current^38^: the generated current is proportional to the X-ray radiation absorbed in the device (which is dependent on the device’s thickness), whilst the saturation current decreases exponentially with bandgap. Although the In_0.5_Ga_0.5_P was thinner than the GaAs device but thicker than the Al_0.52_In_0.48_P photodiode, a higher open circuit voltage was observed with respect to the GaAs device, and a lower open circuit voltage was found with respect to the Al_0.52_In_0.48_P; this shows that the open circuit voltage was mainly influenced by the bandgap.

An almost flat trend was observed for the short circuit current with temperature as is presented in Fig. 5b; these results are in accordance with those reported for an Al_0.52_In_0.48_P ^55^Fe radioisotope microbattery X-ray photovoltaic cell^19^. The number of carriers generated at the absorption of an X-ray photon is expected to have increased at high temperatures due to the likely lower electron-hole pair creation energy; however, this may be compensated by the decrease in the carrier diffusion lengths when the temperature was increased, resulting in the observed flat trend.

**Figure 6:**
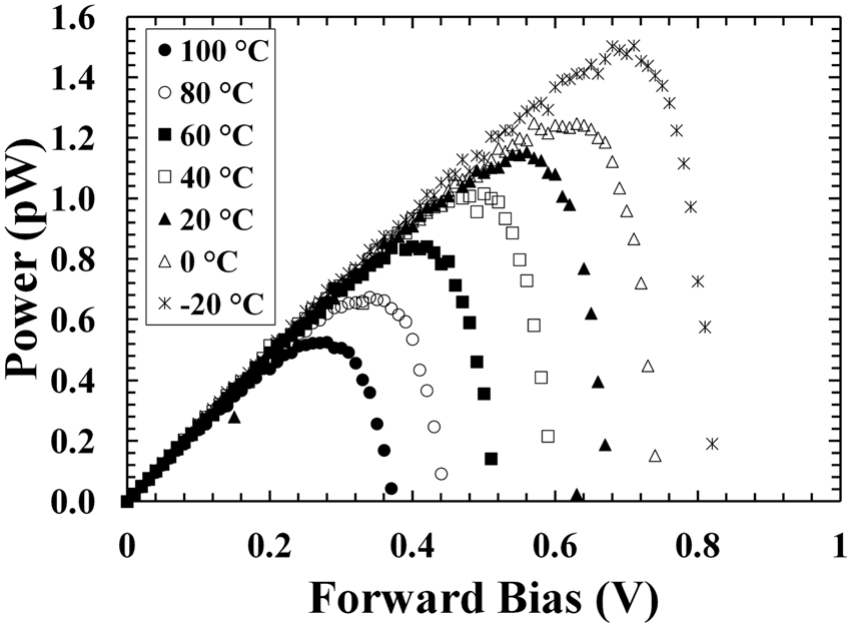
Output power as a function of applied forward bias for the ^55^Fe X-ray photovoltaic In_0.5_Ga_0.5_P cell at different temperatures. The temperatures studied were 100 °C (filled circles), 80 °C (empty circles), 60 °C (filled squares), 40 °C (empty squares), 20 °C (filled squares), 0 °C (empty squares), and −20 °C (stars).

The output power (*P*) extracted from the ^55^Fe X-ray photovoltaic In_0.5_Ga_0.5_P cell is shown in Fig. 6. It was calculated as *P* = *IV*. When the forward bias was increased, the output power increased to a maximum (*P*_*m*_) and then decreased.

**Figure 7:**
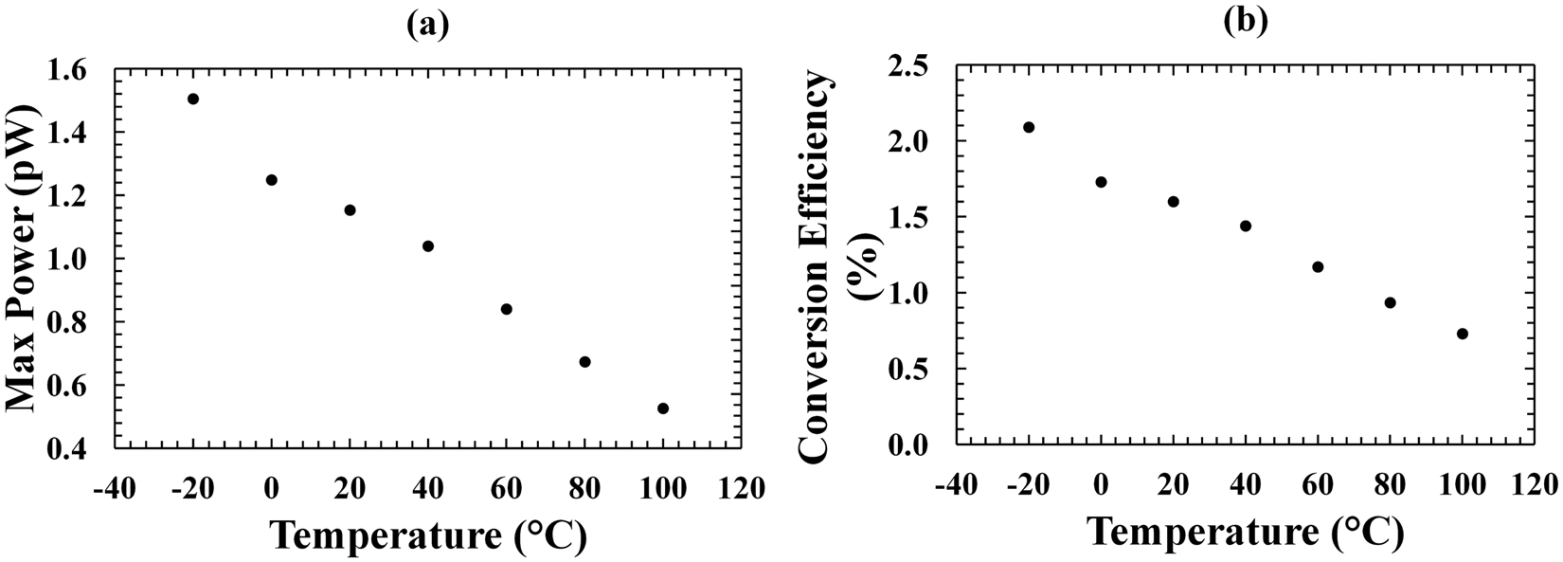
(**a**) Experimental maximum output power as a function of temperature for the ^55^Fe X-ray photovoltaic In_0.5_Ga_0.5_P cell. (**b**) Conversion efficiency (*η*) as a function of temperature for the In_0.5_Ga_0.5_P ^55^Fe X-ray photovoltaic cell.

The maximum output power (*P*_*m*_) as a function of temperature for the ^55^Fe X-ray photovoltaic In_0.5_Ga_0.5_P cell was computed and is presented in Fig. 7a. As expected, *P*_*m*_ increased when the temperature was decreased, this is a consequence of its dependence on the open circuit voltage^38^. The conversion efficiency for the ^55^Fe X-ray photovoltaic In_0.5_Ga_0.5_P cell is shown in Fig. 7b. It was defined as the ratio between *P*_*m*_ and *P*_*i*_ (*P*_*i*_ = 72 pW, calculated in section “B. Theoretical calculations”). The conversion efficiency increased with decreased temperature, and reached its maximum value of 2.1% at −20 °C. Considering the actual power absorbed by the detector (*P*_*th*_ = 7 pW), an internal conversion efficiency (*η*_*i*_ = *P*_*m*_ */ P*_*th*_) of 22% was obtained at −20 °C; the internal conversion efficiency calculation took into account the total quantum efficiency values, *QE*_*Kα*_ and *QE*_*Kβ*_ calculated in section “Device structure and X-ray source”, and the In_0.5_Ga_0.5_P electron-hole pair creation energy (4.8 eV, 2.5 times the bandgap).

A maximimum output power as high as 1.5 pW, corresponding to 0.7 μW/Ci, was extracted from the ^55^Fe X-ray photovoltaic In_0.5_Ga_0.5_P cell at −20 °C. The value of 0.7 μW/Ci was calculated as the ratio between the maximum output power (1.5 pW) and the number of photons expected on the detector (7 × 10^4^ s^−1^ = 2.02 × 10^−6^ Ci, calculated in section “B. Theoretical calculations”). The maximum output power value was higher than those observed by Butera *et al*. using a GaAs ^55^Fe radioisotope microbattery X-ray photovoltaic cell (1 pW)^17^ and an Al_0.52_In_0.48_P ^55^Fe radioisotope microbattery X-ray photovoltaic cell (0.6 pW)^19^, at the same temperature. This was in part due the excellent properties (e.g. high attenuation coefficients, high semiconductor crystalline quality) of the 5 μm i-layer In_0.5_Ga_0.5_P detector. It has to be noted that the GaAs detector used in ref. 17 was 10 μm i-layer thick, whilst the Al_0.52_In_0.48_P device used in ref. 19 was only 2 μm i-layer thick. A comparable output power of 1.3 pW would be expected from a 5 μm i-layer Al_0.52_In_0.48_P device. An increase of the In_0.5_Ga_0.5_P i-layer thickness would further improve the number of X-ray photons absorbed in the detector; such a structure will be considered for the development of a future higher output power In_0.5_Ga_0.5_P cell. For production of a real world In_0.5_Ga_0.5_P microbattery prototype (rather than just analysis of an In_0.5_Ga_0.5_P prototype X-ray photovoltaic cell as reported here), optimisation of the radioisotope X-ray source geometry would also be required since only 0.1% of the emitted photons impinged on the surface of the In_0.5_Ga_0.5_P device in the system used here for evaluating the performance of the In_0.5_Ga_0.5_P cell itself.

## Conclusion

In this paper, for the first time, an In_0.5_Ga_0.5_P ^55^Fe X-ray photovoltaic cell has been reported. A 206 MBq ^55^Fe radioisotope X-ray source was used to illuminate a 400 μm diameter p^+^-i-n^+^ (5 μm i-layer) In_0.5_Ga_0.5_P mesa photodiode to achieve the conversion of X-ray energy into electrical energy. Since In_0.5_Ga_0.5_P is nearly lattice matched with GaAs and can be grown with high crystalline quality, it can be beneficial for the development of microbatteries in many applications. The ^55^Fe radioisotope In_0.5_Ga_0.5_P cell was characterised over the temperature range −20 °C to 100 °C. Preliminary dark current measurements were performed on the In_0.5_Ga_0.5_P detector in order to study the saturation current and ideality factor at different temperatures: both the saturation current and ideality factor decreased between −20 °C and 100 °C. Illuminated current measurements with the ^55^Fe radioisotope In_0.5_Ga_0.5_P cell showed that open circuit voltage, maximum power, and conversion efficiency decreased between −20 °C and 100 °C, whilst an almost flat trend was found for the short circuit current. An open circuit voltage and a conversion efficiency as high as 0.82 V and 2.1%, respectively, were measured at −20 °C. The maximum output power extracted from the ^55^Fe radioisotope In_0.5_Ga_0.5_P cell was 1.5 pW at −20 °C.
